# Melatonin Minimizes the Impact of Oxidative Stress Induced by Hydrogen Peroxide in *Saccharomyces* and Non-conventional Yeast

**DOI:** 10.3389/fmicb.2018.01933

**Published:** 2018-08-20

**Authors:** Jennifer Vázquez, Karlheinz Grillitsch, Günther Daum, Albert Mas, María-Jesús Torija, Gemma Beltran

**Affiliations:** ^1^Oenological Biotechnology Research Group, Department of Biochemistry and Biotechnology, Faculty of Oenology, University of Rovira i Virgili, Tarragona, Spain; ^2^Austrian Centre of Industrial Biotechnology, Graz, Austria; ^3^Institute of Biochemistry, Graz University of Technology, Graz, Austria

**Keywords:** *Torulaspora delbrueckii*, *Metschnikowia pulcherrima*, *Starmerella bacillaris*, *Hanseniaspora uvarum*, ROS, TBARS, catalase, peroxisomes

## Abstract

Melatonin (*N*-acetyl-5-methoxytryptamine) is synthesized from tryptophan by *Saccharomyces cerevisiae* and non-conventional yeast species. Antioxidant properties have been suggested as a possible role of melatonin in a *S. cerevisiae* wine strain. However, the possible antioxidant melatonin effect on non-*Saccharomyces* species and other strains of *S. cerevisiae* must be evaluated. The aim of this study was to determine the antioxidant capacity of melatonin in eight *S. cerevisiae* strains and four non-conventional yeasts (*Torulaspora delbrueckii*, *Metschnikowia pulcherrima*, *Starmerella bacillaris*, and *Hanseniaspora uvarum*). Therefore, the ROS formation, lipid peroxidation, catalase activity, fatty acid composition, and peroxisome proliferation were investigated. The results showed that the presence of melatonin increases peroxisome accumulation and slightly increases the catalase activity. When cells grown in the presence of melatonin were exposed to oxidative stress induced by H_2_O_2_, lower ROS accumulation and lipid peroxidation were observed in all tested strains. Therefore, the increased catalase activity that was a consequence of oxidative stress was lower in the presence of melatonin. Moreover, the presence of MEL modulates cell FA composition, increasing oleic and palmitoleic acids and leading to higher UFA/SFA ratios, which have been previously related to a higher tolerance to H_2_O_2_. These findings demonstrate that melatonin can act as an antioxidant compound in both *S. cerevisiae* and non-*Saccharomyces* yeasts.

## Introduction

Melatonin (*N*-acetyl-5-methoxytryptamine) (MEL) is not only known as a neurohormone in vertebrates, but it is as well considered as a ubiquitous molecule that is present in most living organisms (Hardeland and Poeggeler, 2003). Sprenger et al. (1999) were the first authors to associate the production of MEL with *Saccharomyces cerevisiae*. Later, other reports showed high quantities of MEL being produced by *S. cerevisiae*, and by other non-conventional yeast species such as *Torulaspora delbrueckii* and *Zygosaccharomyces bailii* (Rodriguez-Naranjo et al., 2011; Vigentini et al., 2015). Although only limited information is available on MEL biosynthesis in organisms other than vertebrates, the pathway in yeasts is thought to be similar to the synthetic route described in vertebrates. Four enzymes are involved in the conversion of tryptophan into serotonin and *N*-acetylserotonin intermediates and finally into MEL (Mas et al., 2014).

The functions of MEL have been extensively studied in mammals and animals, and they are primarily related to the regulatory mechanisms involved in circadian rhythms. However, the role of MEL in yeasts still needs to be elucidated. Recently, we have reported that MEL is able to act as antioxidant compound in one commercial wine strain of *S. cerevisiae* (Vázquez et al., 2017). As is the case in humans, MEL might protect various biomolecules from damages caused by free radicals by acting as a direct scavenger, detoxifying reactive oxygen and nitrogen species (Reiter et al., 2001, 2016; Anisimov et al., 2006), and indirectly increasing the activities of antioxidant defense systems. It could also act by stimulating the synthesis of other important intracellular antioxidants such as glutathione peroxidase and superoxide dismutase (Antolín et al., 1996; Rodriguez et al., 2004).

Oxidative stress is the outcome of an imbalance between the presence of reactive oxygen species (ROS) and the capacity of cells to detoxify these reactive intermediates of molecular oxygen, or to repair the resulting damage. ROS are constantly generated during normal metabolism, and they exert physiological actions. However, when produced in excess, ROS cause detrimental effects and can damage cell macromolecules, such as DNA, lipids or proteins (Gutteridge and Halliwell, 2000; Halliwell, 2006). Among these targets of ROS, lipid peroxidation leads to one of the most damaging consequences for cells when unsaturated lipids are converted into polar lipid hydroperoxides because it allows the propagation of free radical reactions that could affect membrane integrity and even result in cell death (Howlett and Avery, 1997; Ayala et al., 2014). However, ROS formation is accompanied by an increase in yeast antioxidant defenses, with the aim of protecting the cells against noxious ROS. One system for neutralizing the excessive ROS formation in cells is to degrade them with antioxidant enzymes, such as catalase, glutathione peroxidase, and superoxide dismutase. By contrast, non-enzymatic systems, such as glutathione, glutaredoxins, and thioredoxins repair or remove the products of oxidative damage (Jamieson, 1998; Costa and Moradas-Ferreira, 2001; Auchère et al., 2008; Herrero et al., 2008).

Due to its high fermentation capacity, *S. cerevisiae* is the yeast that is traditionally used in the biotechnology, food and beverage industries. However, non-*Saccharomyces* yeasts are now gaining higher interests for industries; in fact, several studies have demonstrated that the presence of non-conventional yeasts during winemaking process can contribute to the aroma profile, sensory complexity and color stability (Jolly et al., 2014). During these industrial processes, yeasts are involved in different stages that can lead to oxidative stress for the cells, which could negatively affect yeast performance (Pretorius, 2000; Gómez-Pastor et al., 2012; Pérez-Gallardo et al., 2013). Thus, protective treatments against oxidative damage with natural antioxidants may have important biotechnological implications.

The goal of this study was to evaluate the possible antioxidant effect of MEL on different yeast species. To this end, we evaluated ROS production, lipid peroxidation and intracellular catalase activity in sixteen yeast strains of different species. We evaluated the response to oxidative stress induced by H_2_O_2_ and analyzed the possible protective activity of MEL supplementation.

## Materials and Methods

### Yeast Strains and Experimental Conditions

The yeast strains used in this study were eight *S. cerevisiae* and eight non-*Saccharomyces*. The *S. cerevisiae* strains included three laboratory strains (BY4741, BY4742, and Sigma 1278b from EUROSCARF collection, Frankfurt, Germany), three commercial wine strains [QA23^®^, uvaferm HPS^®^ and the hybrid VIN7 (*S. cerevisiae* x *S. kudriavzevii* AWRI1539^®^)] and two commercial strains for animal nutrition (Levucell^®^ SC20 and SB20). The non-*Saccharomyces* species included two wine strains of *T. delbrueckii* [BIODIVA^®^ (TdB) and Tdp], two wine strains of *Metschnikowia pulcherrima* [FLAVIA^®^ (MpF) and Mpp], two wine strains of *Starmerella bacillaris* (Cz4 and Cz11), and two wine strains of *Hanseniaspora uvarum* (Hu4 and Hu35). The commercial *Saccharomyces* and non-*Saccharomyces* strains QA23, Uvaferm HPS, SC20, SB20, FLAVIA, and BIODIVA were provided by Lallemand S.A. (Montreal, QC, Canada), and VIN7 was provided by AWRI (Glen Osmond, SA, Australia). The other six non-*Saccharomyces* strains (Tdp, Mpp, Cz4, Cz11, Hu4, and Hu35) were isolated from natural musts from Priorat Appellation of Origin (Catalonia, Spain) (Padilla et al., 2016). The Tdp, Mpp, Cz4, and Hu4 were deposited in the Spanish Type Culture Collection (CECT) as CECT 13135, CECT 13131, CECT 13129, and CECT 13130, respectively.

All commercial strains were provided as active dry yeast and rehydrated according to the manufacturer’s instructions. For all experiments, precultures for biomass propagation were prepared in YPD liquid medium [2% (w/v) glucose, 2% (w/v) peptone, and 1% (w/v) yeast extract (PanReac, Barcelona, Spain)] and incubated for 24 h at 28°C with orbital shaking (120 rpm). Yeast cells were subsequently inoculated into 50 mL of YPD broth (initial population 5 × 10^3^ cells/mL) with and without supplementation of 5 μM MEL (two flasks for each condition) and grown until cells reached the initial exponential phase at 28°C with orbital shaking at 120 rpm. Sublethal oxidative stress was then induced in one flask of each condition with 2 mM of H_2_O_2_ for 1 h to generate the following four conditions: Control and MEL (without stress); and H_2_O_2_ and MEL H_2_O_2_ (with stress). The MEL and H_2_O_2_ concentrations were chosen from our previous study in the QA23 strain (Vázquez et al., 2017). Three biological replicates were tested for each condition.

### Determination of Reactive Oxygen Species (ROS)

The effect of H_2_O_2_ (2 mM) with and without MEL (5 μM) on the intracellular ROS was evaluated in the sixteen yeast strains. Furthermore, ascorbic acid (25 μM), a well-known antioxidant, was used as positive control (Saffi et al., 2006). ROS was detected using dihydrorhodamine 123 (DHR 123; Sigma-Aldrich), according to the method used by Vázquez et al. (2017). In brief, the cells were stained with 10 μg DHR 123 per mL of cell culture for 15 min at 120 rpm in the dark. Cells were then washed twice with phosphate-buffered saline (PBS, pH 7.4), and the fluorescence intensity was measured by flow cytometry. The captured files were processed using WinMDI 2.9 software (Joseph Trotter, Salk Institute for Biological Studies, La Jolla, CA, United States) and the ROS were represented as the mean fluorescence index (MFI) and calculated according to Boettiger et al. (2001) as follows: [(geometric mean of the positive fluorescence) – (geometric mean of the control)]/(geometric mean of the control).

### Thiobarbituric Acid Reacting Substances (TBARS)

The degree of lipid peroxidation was measured in unstressed and stressed cells with and without MEL supplementation in terms of TBARS content (Buege and Aust, 1978; Aust, 1994). Following a treatment using 2 mM of H_2_O_2_ for 1 h, 1 × 10^7^ yeast cells from each condition were mechanically homogenized over three cycles of alternating sonication and liquid nitrogen (10/10 s). The samples were then mixed with 250 μL of trichloroacetic acid (10% v/v), incubated 15 min on ice, and after centrifugation at 2200 *g* for 15 min at 4°C, 200 μL of the supernatant was mixed with 200 μL thiobarbituric acid (6.7 g/L). These samples were then incubated in a boiling water bath for 10 min and cooled at room temperature. Finally, the absorbance was measured at 532 nm with a microplate reader (Omega POLARstar, BMG LABTECH GmbH, Ortenberg, Germany). The TBARS content was estimated by referring to a standard curve prepared with 1,1,3,3-tetramethoxypropane and the results were expressed as nmol of TBARS per mg of protein.

### Catalase Activity

The catalase activity was evaluated in unstressed and stressed cells with and without MEL. First, 1 × 10^7^ yeast cells were suspended in PBS (50 mM, pH 7.0) with one tablet of protease inhibitor per 10 mL of extraction solution (cOmplete^TM^; Roche), and they were disrupted using glass beads with six cycles alternating cooling and shaking (30/30 s) and centrifuged at 14.000 rpm for 2 min. The assay was performed according to the method described by Aebi (1984). In brief, cells extracts were exposed to 10 mM of H_2_O_2_, and the decrease in absorbance at 240 nm due to H_2_O_2_ decomposition was monitored for 4 min, with measurements every 30 s at constant temperature (25°C) using a microplate reader (Omega POLARstar, BMG LABTECH GmbH, Ortenberg, Germany). The catalase activity was expressed as units of catalase per mg of protein. One unit of catalase activity decomposes 1 mmol of H_2_O_2_ per min.

### Protein Estimation

The total protein levels were determined using the Bradford method (Bradford, 1976) by spectrophotometric determination at 545 nm, with bovine serum albumin (BSA, Sigma-Aldrich) as a standard. The absorbance was measured in an Omega POLARstar microplate reader spectrophotometer.

### Yeast Viability After Stress Exposure

As in our previous study with *S. cerevisiae* (Vázquez et al., 2017), the viability of non-*Saccharomyces* strains after exposure to stress (MEL H_2_O_2_, ASC H_2_O_2_, and H_2_O_2_) in comparison with cells without stress (Control and MEL) was evaluated by a microplate assay. 96-well plates were prepared by dispensing 250 μL of YPD broth inoculated with cells of each condition to obtain an initial OD_600_ of 0.005. The microplate was incubated at 28°C and the optical density at 600 nm was measured every 30 min during 30 h using a microplate reader (Omega POLARstar, BMG LABTECH GmbH, Ortenberg, Germany).

### Analysis of Fatty Acid Composition

The composition of fatty acids (FAs) of QA23 and TdB strains was analyzed in cells which were untreated and treated with 5 μM of MEL in absence of stress (Control and MEL) and 18 h after the oxidative stress (2 mM H_2_O_2_) was applied to allow the cells to respond to this stress (MEL H_2_O_2_ and H_2_O_2_). Yeast cells homogenates were obtained from 50 mL of total cells pellets using glass beads and a Disruptor Genie^®^ (Scientific Industries, Inc., NY, United States) for 10 min at 4°C. Proteins from the homogenates were then precipitated with 10% (v/v) trichloroacetic acid and quantified with de Folin phenol reagent (Lowry et al., 1951). The total lipids were extracted from cell fractions corresponding to 1 mg of total cell protein according to the method by Folch et al. (1957). The FA composition was determined by gas liquid chromatography (GLC) according to Rußmayer et al. (2015). In brief, the total FAs from lipid extracts were first converted to methyl esters by methanolysis with sulfuric acid (2.5% in methanol (v/v); 80°C for 90 min) and then extracted twice with light petroleum and water (3:1; v/v) by shaking on a Vibrax orbital shaker^®^ (IKA, Staufen, Germany) for 30 min. Finally, FAs were separated by GLC on a Hewlett-Packard 6890 gas-chromatograph (Agilent Technologies, CA, United States) using an HP-INNOWax capillary column (15 m × 0.25 mm × 0.50 μm film thickness) with helium as a carrier gas. Identification was done by comparison with a commercial FA methyl ester standard mix (NuCheck, Inc., MN, United States) and quantification using the pentadecanoic acid (C15:0, Sigma-Aldrich) as an internal standard. Two biological replicates were set up for each strain and two analytical replicates were performed for each biological replicate.

### Western Blot (Immunoblot) Analysis

The immunological characterization of QA23 and TdB strain homogenates from the four conditions (Control, MEL, MEL H_2_O_2_, and H_2_O_2_) was performed by Western blot analysis as described by Haid and Suissa (1983). In brief, the cells were disrupted with glass beads using a Disruptor Genie^®^ (Scientific Industries, Inc., NY, United States) at 4°C for 10 min and centrifuged at 4°C at 500 *g* for 5 min. After TCA precipitation of supernatants, protein samples were separated by sodium dodecyl sulfate polyacrylamide gel electrophoresis (SDS-PAGE, 12.5%), and later transferred to the nitrocellulose sheets according to standard procedures (Laemmli, 1970). Finally, a western blot analysis was performed using a primary rabbit antibody against Fox1p protein (multifunctional β-oxidation protein from peroxisomal membranes), a marker of peroxisomes organelles. Immunoreactive bands were visualized using a peroxidase-conjugated secondary antibody according to the manufacturer’s instructions (SuperSignal^TM^, Pierce Chemical Company, IL, United States). The cytosolic protein GAPDH (glyceraldehyde-3-phosphate dehydrogenase) was used as a loading control, and isolated peroxisomes from *S. cerevisiae* were the positive control. The identified bands were quantified using ImageJ software (National Institutes of Health, MD, United States) and normalized to positive control.

### Data Analysis

The data were subjected to one-way analysis of variance (ANOVA) and Tukey’s *post hoc* test to evaluate the effect of each treatment. The results were considered statistically significant at a *p*-value less than 0.05 (IBM SPSS Inc, XLSTAT Software).

## Results

### Reactive Oxygen Species (ROS)

**FIGURE 1:**
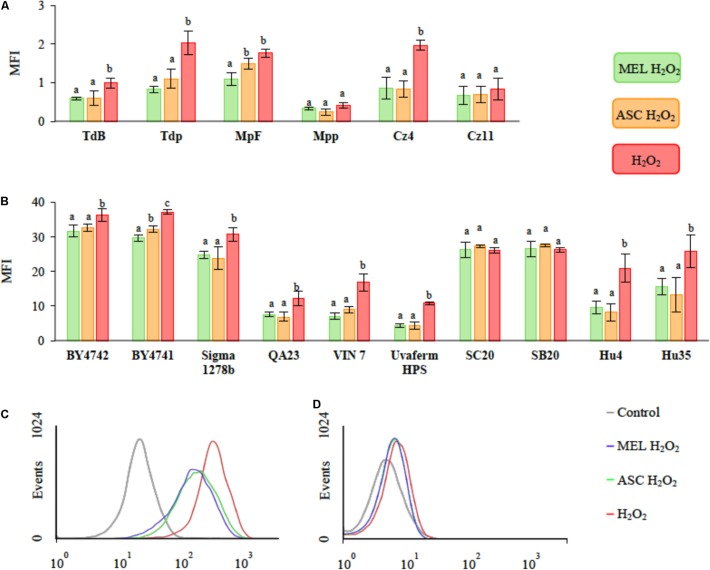
Melatonin (MEL, 5 μM) effect on ROS accumulation as evaluated in sixteen yeast strains under oxidative stress that was induced with 2 mM of H_2_O_2_. Cells without MEL and H_2_O_2_ were used as negative control, cells with 2 mM of H_2_O_2_ were used as the positive control and cells treated with ascorbic acid (25 μM) served as the positive antioxidant control. **(A)** The mean fluorescence index (MFI) of the *T. delbrueckii* (TdB and Tdp), *M. pulcherrima* (MpF and Mpp), and *C. zemplinina* (Cz4 and Cz11) strains. **(B)** The MFI of the *S. cerevisiae* (BY4742, BY4741, Sigma 1278b, QA23, VIN 7, SC20, SB20, and Uvaferm HPS) and *H. uvarum* (Hu4 and Hu35) strains. **(A,B)** Different letters indicate significant differences between conditions within each strain, *p* < 0.05. **(C,D)** Flow cytometry histogram profiles expressed as the number of events in VIN 7 **(C)** and Cz4 **(D)** with MEL (5 μM) or ascorbic acid (ASC, 25 μM) and with 2 mM of H_2_O_2_.

To evaluate the possible role of MEL as an antioxidant agent in the *Saccharomyces* and non-*Saccharomyces* species, the intracellular ROS levels were measured in stressed cells with and without 5 μM of MEL (Vázquez et al., 2017). Stressed cells with 2 mM of H_2_O_2_ and without MEL were used as positive control and cells without stress and without MEL were used as a negative control. Cells treated with 25 μM of ascorbic acid were used as positive antioxidant control (**Figure 1**). The results showed that cells that had been exposed to oxidative stress (2 mM H_2_O_2_) exhibited an increase in the total ROS. However, the ROS accumulation was species-dependent, with *M. pulcherrima*, *S. bacillaris* and *T. delbrueckii* exhibiting the lowest levels of endogenous ROS (**Figures 1A,D**). By contrast, the *Saccharomyces* and *H. uvarum* strains presented the highest levels of ROS (**Figures 1B,C**). For the *S. cerevisiae* strains, clear differences were observed between the wine strains and laboratory and animal nutrition strains, with the wine strains exhibiting the lower levels of endogenous ROS. The antioxidant effects of MEL were very similar to those of ascorbic acid for most investigated strains. However, there were few cases in which none of the antioxidants had any protective effect (*S. cerevisiae* strains SC20 and SB20, *M. pulcherrima* Mpp and *S. bacillaris* Cz11 (**Figure 1**).

### Lipid Peroxidation

**FIGURE 2:**
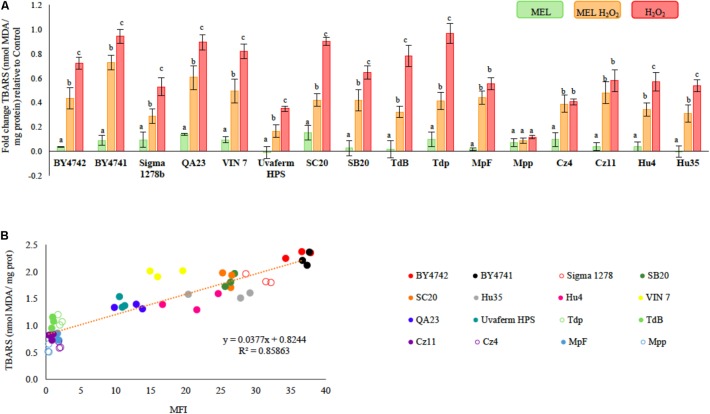
**(A)** Lipid peroxidation in unstressed and stressed yeast cells with 2 mM of H_2_O_2_, growing with and without 5 μM of melatonin (MEL). The strains analyzed (*n* = 3 of each strain) were *S. cerevisiae* (BY4742, BY4741, Sigma 1278b, QA23, VIN 7, SC20, SB20, and uvaferm HPS), *T. delbrueckii* (TdB and Tdp), *M. pulcherrima* (MpF and Mpp), *C. zemplinina* (Cz4 and Cz11), and *H. uvarum* (Hu4 and Hu35). Error bars represent ± SD of *n* = 3 by ANOVA. Different letters indicate significant differences between conditions within each strain, *p* < 0.05. **(B)** The correlation between ROS accumulation (MFI, **Figure 1**) and lipid peroxidation (TBARS, **Supplementary Table S1**) in the sixteen strains (*n* = 3 of each strain) after stress exposure with H_2_O_2_ (2 mM).

The effect of MEL on oxidative damage in the membranes was evaluated in all yeasts by measuring the lipid peroxides in the TBA derivative form (**Figure 2A**). Most strains studied here suffered from a significant increase in lipid peroxidation after stress exposure, with the Mpp strain being the only one in which its lipid peroxidation was not affected by H_2_O_2_. In fact, the lipid peroxidation results were positively correlated with ROS accumulation (**Figure 2B**, *R*^2^ = 0.85863). Thus, the *M. pulcherrima*, *S. bacillaris*, and *T. delbrueckii* strains, which showed lower ROS accumulation, also exhibited lower lipid peroxidation and vice versa. Strains with higher ROS accumulation showed higher lipid peroxidation (**Figure 2B**). The positive effect of MEL supplementation was clearly observed under stress conditions (**Figure 2A**, MEL H_2_O_2_), in which MEL seems to protect *Saccharomyces*, *T. delbrueckii*, and *H. uvarum* cells against H_2_O_2_ damage by decreasing lipid peroxidation. In *M. pulcherrima* and *S. bacillaris*, no MEL effect was observed on lipid peroxidation. However, no significant differences were observed in the lipid peroxidation between unstressed cells with or without MEL, although in some strains, there was an increasing trend in lipid peroxidation in presence of MEL (**Figure 2A** and **Supplementary Table S1**, Control and MEL).

### Catalase Activity

**FIGURE 3:**
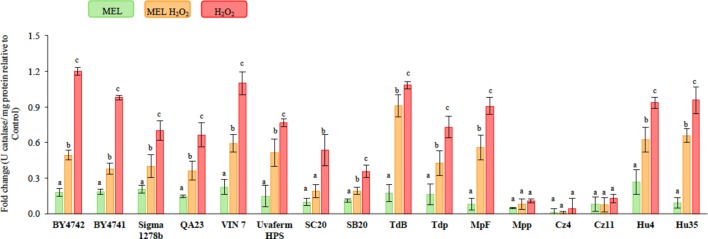
Catalase activity in unstressed and stressed yeast cells with 2 mM of H_2_O_2_, growing with and without 5 μM of melatonin (MEL). The strains used were: *S. cerevisiae* (BY4742, BY4741, Sigma 1278b, QA23, VIN 7, SC20, SB20, and uvaferm HPS), *T. delbrueckii* (TdB and Tdp), *M. pulcherrima* (MpF and Mpp), *C. zemplinina* (Cz4 and Cz11), and H. uvarum (Hu4 and Hu35). The error bars represent ± SD of *n* = 3 by ANOVA. Different letters indicate significant differences between conditions within each strain, *p* < 0.05.

To further study the role of MEL in yeasts, the effect of its supplementation (5 μM MEL) on catalase activity was evaluated in unstressed and stressed cells (**Figure 3**). The control condition (without stress and without MEL) for non-conventional yeasts showed higher catalase activity than did the *Saccharomyces* species (**Supplementary Table S1**). When MEL was added in absence of stress, the catalase activity of *Saccharomyces*, *T. delbrueckii*, and *H. uvarum* slightly increased (**Figure 3** and **Supplementary Table S1**). However, when cells were exposed to H_2_O_2_, the catalase activity clearly increased in all the strains except for Mpp and *S. bacillaris* (**Figure 3**). However, this activity was significantly reduced when the cells had been grown in the presence of MEL before the stress was applied (**Figure 3** and **Supplementary Table S1**). Under these stress conditions, no numeric correlation was found between the catalase activity and ROS accumulation or TBARS assay (data not shown).

### Effect of Melatonin on Cell Growth Viability After Oxidative Stress Exposure

**FIGURE 4:**
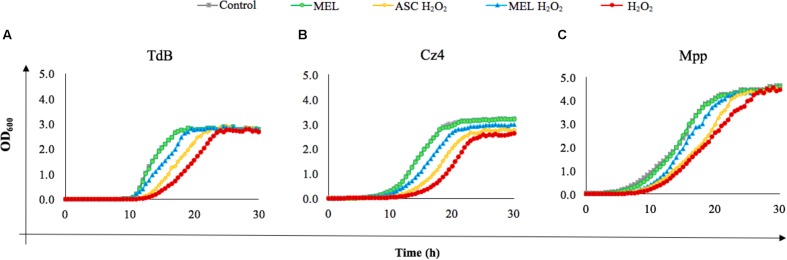
Melatonin effect on cell growth viability after oxidative stress exposure (2 mM of H_2_O_2_) in three non-*Saccharomyces* yeast (**A**, *T. delbrueckii* TdB strain; **B**, *S. bacillaris* Cz4 strain; and **C**, *M. pulcherrima* Mpp strain). Stress was applied in early exponential phase. H_2_O_2_, stressed cells; MEL H_2_O_2_, stressed cells in presence of 5 μM of MEL; ASC H_2_O_2_, stressed cells in presence of 25 μM of ascorbic acid. Two conditions were considered as controls. Control, unstressed cells; MEL, unstressed cells in presence of 5 μM of MEL.

To evaluate the effect of MEL on the growth of non-*Saccharomyces* strains after stress exposure, cells were reinoculated in YPD, and growth was followed during 30 h (**Figure 4**). In the absence of stress, similar growth curves were observed for both conditions (with and without MEL). In contrast, under oxidative stress, viability of all strains was significantly affected; the presence of MEL was able to greatly enhance cell growth in non-*Saccharomyces* strains, recovering, in general, a growth curve similar to non-stressed cells. In addition, the effect of 5 μM of MEL was higher than that of 25 μM of ascorbic acid.

### Changes in FA Composition

**FIGURE 5:**
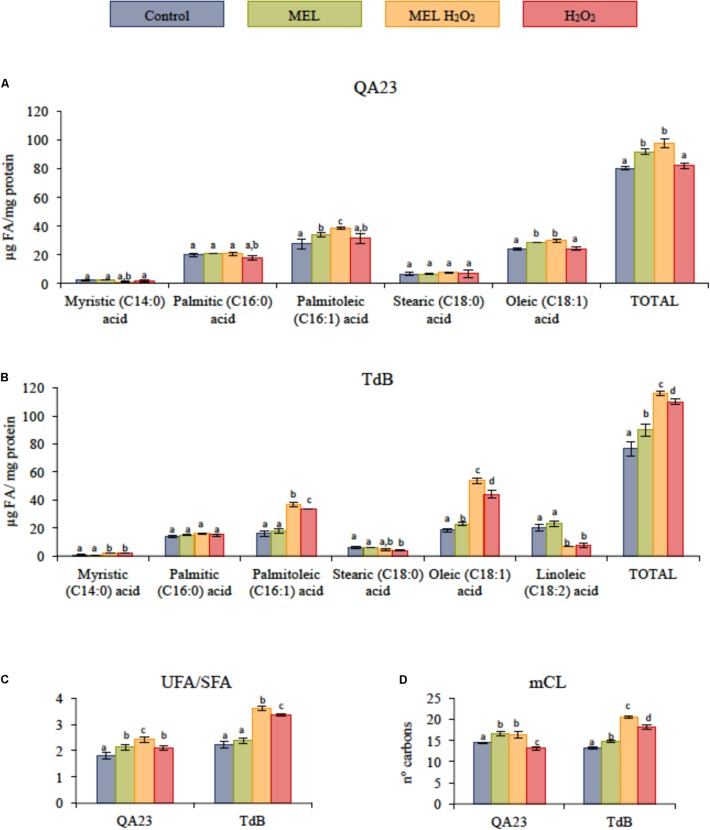
Fatty acid (FA) composition of unstressed and stressed yeast cells with 2 mM of H_2_O_2_, growing with and without 5 μM of melatonin (MEL). **(A)** FA of QA23 strain. **(B)** FA of TdB strain. **(C)** Unsaturated to saturated FA ratio (UFA/SFA) of both strains. UFA/SFA ratio was defined as follows: (C16:1 + C18:1 + C18:2)/(C14:0 + C16:0 + C18:0). **(D)** Medium length chain of FA of both strains. mCL was calculated as follows: Σ(%FA × N° FA carbons)/100. Different letters in superscripts indicate significant differences between conditions for each studied compound **(A,B)** or for each strain **(C,D)** (*P* < 0.05).

The effect of MEL on FA content under the four experimental conditions (Control, MEL, MEL H_2_O_2_, and H_2_O_2_, **Figure 5**) was tested in two strains (QA23 and TdB). In general, the total FA content increased mainly due to a higher content of monounsaturated fatty acids (MUFAs), such as oleic and palmitoleic acids (**Figures 5A,B**). In *S. cerevisiae* QA23 strain, the highest content of FA was found under melatonin conditions, whereas in the TdB strain, under stress conditions. Similar results were observed in unsaturated to saturated FA ratio (UFA/SFA) and percentage of medium chain length (mCL), being the MEL H_2_O_2_, the condition with the highest UFA/SFA ratio in both strains (**Figures 5C,D**). On the other hand, TdB strain presented also linoleic acid in its lipid composition, which significantly decreased after stress exposure.

### Analysis of Peroxisome Proliferation

**FIGURE 6:**
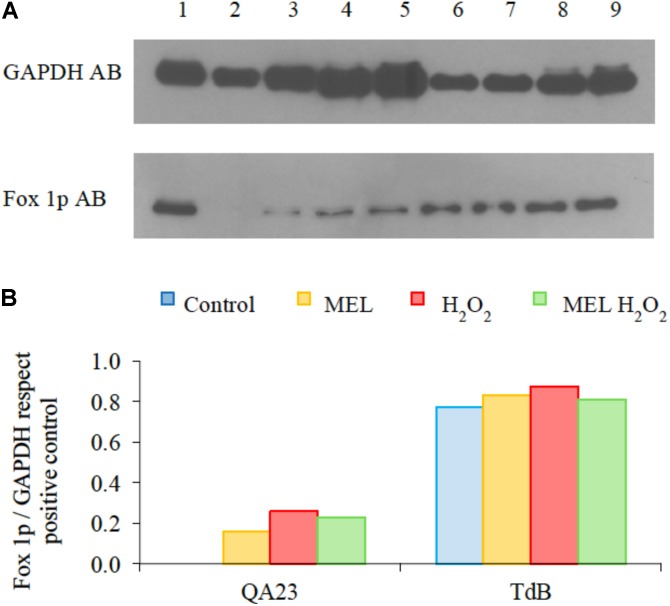
Western blot analysis of homogenates analyzed with antibody against peroxisome marker Fox1p using antibody GAPDH antibody as the loaded protein control. **(A)** Isolated peroxisomes from *S. cerevisiae* were used as the positive control (1). Homogenates from *S. cerevisiae* QA23 (2–5) and *T. delbrueckii* TdB (6–9) in cells without treatment (Control: 2 and 6), cells without stress and 5 μM of melatonin (MEL, 3 and 7), cells with 2 mM H_2_O_2_ (4 and 8) and cells with 2 mM H_2_O_2_ and 5 μM of MEL (5 and 9). **(B)** Fox1p/GAPDH quantification from the bands identified in **A** was normalized to the positive control.

A western blot analysis using the direct antibody against Fox1p, a multifunctional β-oxidation protein from the peroxisomal membranes, was performed with QA23 and TdB homogenates, with and without stress exposure, and in the presence or absence of MEL. As shown in **Figures 6A,B**, the enrichment of Fox1p was higher in *T. delbrueckii* than in *S. cerevisiae* under both stressed and unstressed conditions. Under the control condition, Fox1p was undetectable in *S. cerevisiae*, but its detection increased in the presence of H_2_O_2_. Instead, *T. delbrueckii* showed a high number of peroxisomes independent of stress exposure. MEL induced the proliferation of peroxisomes in the absence of stress, especially in *S. cerevisiae*. Under stress conditions, MEL seemed to decrease the peroxisomes accumulation slightly in both species.

## Discussion

The role of MEL in cells has been extensively studied in humans and other organisms (Hardeland and Poeggeler, 2003; Tan et al., 2015) and its antioxidant capacity is one of the most important biological activities described to date. *S. cerevisiae* synthetizes MEL from tryptophan during alcoholic fermentation (Mas et al., 2014), but very little information is available on MEL biosynthesis and its bioactive functions in yeast. Recently, we reported that MEL is able to act as an antioxidant compound in a wine *S. cerevisiae* strain (Vázquez et al., 2017); however, its antioxidant role in other *Saccharomyces* strains and other non-conventional yeast species is still unknown. Therefore, sixteen strains from five different yeast species were used to evaluate if the protective effect of MEL against oxidative stress is provided due to intra or interspecific response.

As expected, ROS formation was positively correlated with lipid peroxidation. Exposure to oxidative stress has been reported to cause an increase in intracellular ROS that resulted in a loss of membrane integrity due to the peroxidation of unsaturated fatty acids by ROS because the polyunsaturated fatty acids (PUFAs) are more prone to oxidation than MUFAs (Ayala et al., 2014; Johansson et al., 2016). However, in our study, the non-*Saccharomyces* species that include PUFAs as native constituent in their biological membranes (*T. delbrueckii*, *M. pulcherrima*, and *S. bacillaris*) (Rozès et al., 1992), have exhibited higher resistance to oxidative stress together with lower ROS formation and lower lipid peroxidation. Cipak et al. (2008) reported that even if a PUFA-producing *S. cerevisiae* yeast was initially more sensitive to oxidative stress than the wild-type strain, this transgenic strain became more resistant to H_2_O_2_ after some time of cultivation had passed, indicating that there was an adaptation to the endogenous oxidative stress due to the presence of PUFAs. The authors hypothesized that the presence of those PUFAs during aerobic growth generated low but significant levels of lipid peroxidation products (specifically 4-hydroxynonenal, or HNE), even in the absence of exogenous stress, which can act as a signaling molecule to activate the stress response and prepare the cells for subsequent stresses (Chen et al., 2006; Cipak et al., 2008). At sublethal concentrations, the accumulation of lipid peroxidation products stimulates the defense network, triggering the early response enzymes (antioxidative and detoxifying enzymes) and induces an adaptive response to cope with the forthcoming oxidative stress (Chen et al., 2006). A similar stress response mechanism might explain the higher resistance to oxidative stress of these yeast species of our study that characteristically contained membranes rich in PUFAs. Nevertheless, a decrease of PUFAs and an increase of MUFAs after stress were observed in these species, which could be a mechanism of non-*Saccharomyces* yeasts to better resist oxidative stress without compromising membrane integrity.

The results obtained here show that under unfavorable conditions that affect the redox balance, *Saccharomyces*, *T. delbrueckii*, and *H. uvarum* clearly take advantage of MEL supplementation in the growth medium, reducing the toxic effects of H_2_O_2_ (decreasing the ROS levels and lipid peroxidation). These results are in accordance with several studies in humans (Tesoriere et al., 1999; Taysi et al., 2003; Ündeğer et al., 2004) and with our previous studies with a wine *S. cerevisiae* strain (Vázquez et al., 2017) in which the protective action of MEL might be attributed to its ability to scavenge ROS particles and consequently prevent cellular damage. MEL is able to act as a direct free radical scavenger and as an indirect antioxidant, detoxifying for numerous ROS including H_2_O_2_, hydroxyl radical (^⋅^OH), peroxyl radicals (ROO^⋅^), singlet oxygen (^1^O_2_), and also reactive nitrogen species (RNS) (Romero et al., 2014). Moreover, MEL was able to enhance cell recovery in non-*Saccharomyces* strains after being exposed to oxidative stress in early exponential phase, while this effect was observed in late exponential phase for QA23 strain in a previous study (Vázquez et al., 2017). This early improvement of cell viability after stress in non-*Saccharomyces* strains could be related to the different lipid composition of their membranes compared to *S. cerevisiae* strains (submitted manuscript). Indeed, the presence of MEL in stressed cells increased the total FA levels, specifically, the MUFAs, leading to higher UFA/SFA ratios, which have been previously related to a higher tolerance to H_2_O_2_ (Serrazanetti et al., 2015). Oleic acid has been reported as a membrane fluidity sensor, and it seems to be the most important UFA for counteracting the toxic nature of ethanol by increasing the membrane stability and antagonizing the fluidity caused by ethanol (You et al., 2003). Furthermore, palmitoleic acid is induced by stress in high-density fermentations, and it has a protective function against damage (Mannazzu et al., 2008; Ding et al., 2009).

Catalases are clearly important for proper resistance toward H_2_O_2._ However, the role of catalases enzymes in yeasts is not fully understood. Catalase A is located in the peroxisome and is primarily responsible for detoxifying H_2_O_2_ formed by acyl-CoA oxidase during β-oxidation, whereas the physiological role of the cytosolic catalase T is less clear. However, the expression of *CTT1* gene, which encodes this enzyme, is regulated by oxidative and osmotic stress (Jamieson, 1998; Krantz et al., 2004). The process of β-oxidation is exclusively housed by peroxisomes in yeast. Here, peroxisomal oxidases, such as Pox1p/Fox1p pass electrons directly to oxygen to generate H_2_O_2_, which is decomposed into water and oxygen by catalase A with concomitant release of energy as heat. β-oxidation per se does not depend on a functional peroxisomal catalase (Hiltunen et al., 2003).

Non-conventional yeasts showed slightly higher catalase activity than *Saccharomyces* strains under the control condition (without stress or MEL). Cipak et al. (2008) uncovered related PUFA production with an increase in the catalase activity, pinpointing cytosolic catalase T as essential for the survival of cells against oxidative stress, and peroxisomal catalase A as important for adapting to this stress. Therefore, higher catalase activity in non-*Saccharomyces* strains prior to stress occurs can also be induced as a response to the presence of PUFA in the membrane composition, resulting in a faster adaptation and a better tolerance to the stress. Although the catalase activity increased in the presence of oxidative stress with H_2_O_2_, no direct correlation between catalase activity and ROS or lipid peroxidation was observed in our results, suggesting that catalase, which is a primary enzymatic defense, is quickly activated in presence of H_2_O_2_ with the aim of avoiding cellular damage neutralizing ROS. Furthermore, this finding could indicate that other antioxidant primary defenses such as superoxide dismutase and glutathione peroxidase (no determined in this study), which rapidly sense and respond to oxidative stress, may also be contributing to the maintainance of the ROS concentrations at a basal level (Jamieson, 1998; Costa and Moradas-Ferreira, 2001; Moradas-Ferreira and Costa, 2013).

Moreover, MEL supplementation increased catalase activity in the *Saccharomyces*, *T. delbrueckii*, and *H. uvarum* strains. Together with our previous results in the QA23 strain (Vázquez et al., 2017) in which we also observed that MEL slightly increased the ROS amount as well as the mRNA levels of *CTT1* and *CTA1* (genes encoding catalase T and catalase A, respectively) and other enzymes involved in primary defense, these current results seem to confirm the role of MEL as a prooxidant that prepares the cells to better endure subsequent stress. As expected, the catalase activity was even higher in cells exposed to H_2_O_2_. When cells exposed to H_2_O_2_ were pretreated with MEL, catalase activity significantly decreased. Similar results were obtained by Saffi et al. (2006), but using L-ascorbic acid as an antioxidant and paraquat as an oxidative agent. The authors hypothesized that the reduced catalase activity caused by the presence of L-ascorbic acid could indicate that L-ascorbic acid has sequestered part of the ROS generated by paraquat, thereby reducing the need for catalase biosynthesis. Therefore, the presence of antioxidant compounds such as MEL would reduce the amount of ROS when an oxidative stress is applied and would modulate the catalase levels in yeast cells.

Peroxisomes play important roles in yeast metabolism, mostly in the β-oxidation of fatty acids and in the degradation of toxic hydrogen peroxide via catalase and other antioxidant enzymes (Hiltunen et al., 2003; Schrader and Fahimi, 2006). The amount of peroxisomes in the cell (proliferation or degradation) is modulated in response to nutritional and environmental stimuli. Our results showed higher peroxisome proliferation in cells under stress coinciding with higher catalase activities, indicating a direct relationship between both parameters. In fact, the responses to oxidative stress in *S. cerevisiae* seem to be co-regulated, similar to the increase of ROS and lipid peroxidation, which activates the proliferation of peroxisomes. The observed increase in the peroxisome proliferation comes hand with elevated catalase activity. To shed cellular organelles from harmful ROS, yeasts sequester ROS in peroxisomes, an organelle specialized and perfectly enzymatically equipped for detoxification of harmful molecules, such as H_2_O_2_. In fact, peroxisomes are considered a source of oxidative stress due to the generation of ROS in its respiratory pathway. However, peroxisomes can also respond to oxidative stress and ROS when they are generated at other intra- or extracellular locations, protecting the cell against oxidative damage (Schrader and Fahimi, 2006). Higher amounts of peroxisomes were observed in TdB strain (together with higher amounts of catalase activity and lower ROS levels), in comparison to QA23. Although several authors have described *T. delbrueckii* as Crabtree positive, its respiratory metabolism makes greater contribution to the overall metabolism than in *Saccharomyces* (Alves-Araújo et al., 2007; Merico et al., 2007). Moreover, genes encoding for peroxisomal β-oxidation in *S. cerevisiae* are repressed by glucose, even in the presence of both oleate and oxygen, which are two inducers of the peroxisomes proliferation (Hiltunen et al., 2003; Schrader and Fahimi, 2006). Therefore, this higher peroxisomal activity of TdB strain, even before stress, together with the lower levels of ROS, indicates that *T. delbrueckii* (TdB strain) could have established a sophisticated strategy to maintain an equilibrium between the production and scavenging of ROS. Peroxisome proliferation was induced by MEL, even without stress and primarily in *S. cerevisiae* (QA23 strain). Those results suggest a possible role of MEL as pro-oxidant because it seems capable to prepare the cells to better endure a later oxidative stress, as observed by Vázquez et al. (2017).

Our results indicate that MEL presents antioxidant properties against hydrogen peroxide stress in all the studied yeasts. To the best of our knowledge, the antioxidant effect of MEL in non-*Saccharomyces* yeasts was not previously investigated. Furthermore, in terms of antioxidant properties, MEL is comparable to vitamin C (Reiter et al., 2007), and its effect was even higher under our conditions, because after stress exposure, cell viability was higher and ROS reduction similar with MEL than with ascorbic acid, but at lower MEL concentration.

The knowledge of the role of MEL in yeast will help to understand its synthesis and to obtain MEL-overproducing strains, which could have important biotechnological implications, such as diminishing cellular oxidative stress during the biotechnological production of yeast starters (Gamero-Sandemetrio et al., 2015). Moreover, a better characterization of this antioxidant mechanism could favor its use as potential therapeutic target for several oxidative stress-related diseases (Halliwell, 2006; Gutteridge and Halliwell, 2010; Escoté et al., 2012).

## Conclusion

In conclusion, the present results provide a significant advance in our understanding of the *in vivo* antioxidant activity of MEL in *Saccharomyces* and non-*Saccharomyces* species. MEL can serve to mitigate oxidative stress and oxidative damage by leading to a decrease in the intracellular ROS content and lipid peroxidation under unfavorable conditions. Furthermore, MEL previously activated catalase activity, reducing the need of its biosynthesis against future oxidative redox changes. Therefore, MEL could be acting at different levels in yeast to reduce the oxidative stress damage: (1) as an antioxidant that directly scavenges ROS, (2) indirectly stimulating the antioxidant enzyme production, (3) by modulating FA composition of membranes, and (4) by increasing the effectiveness of peroxisome functions, which would further decrease lipid peroxidation. Thus, protective treatment with MEL could minimize the oxidative stress suffered by active dry yeast during the biomass propagation and dehydration but also increase the replicative lifespan of yeasts, particularly important in re-pitching practices. On the other hand, MEL synthesis by yeast during wine production could confer cells higher ability to adapt and endure the hostile environment of the winemaking process (low dissolved oxygen concentration, low pH, high osmolarity, ethanol toxicity, nutrient starvation, and non-optimal temperature) and counteract the oxidant effects of ethanol. Thus, the effect of MEL on yeast under stresses present in wine or yeast biomass production should be elucidated.

## Author Contributions

JV designed, performed, and analyzed the experiments, discussed the results, and wrote the manuscript. KG and GD helped in the design and discussion of the peroxisome proliferation analysis and the revision of the manuscript. AM, GB, and M-JT designed the experiments, discussed the results, and wrote the manuscript.

## Conflict of Interest Statement

The authors declare that the research was conducted in the absence of any commercial or financial relationships that could be construed as a potential conflict of interest.
