# Supplementary material for: Melatonin Minimizes the Impact of Oxidative Stress Induced by Hydrogen Peroxide in Saccharomyces and Non-conventional Yeast
Source: Front Microbiol. 2018 Aug 20;9:1933. doi: 10.3389/fmicb.2018.01933 (PMC6109679; doi:10.3389/fmicb.2018.01933)
Supplement: Supplementary file 1 [file Table_1.DOCX]

Supplementary Material

Melatonin minimizes the impact of oxidative stress induced by hydrogen peroxide in *Saccharomyces* and non-conventional yeast

Jennifer Vázquez^1^, Karlheinz Grillitsch^3^, Günther Daum^2^, Albert Mas^1^, María Jesús Torija^1^*, Gemma Beltran^1^

*** Correspondence:** Corresponding Author: [mjesus.torija@urv.cat](mailto:mjesus.torija@urv.cat)

**Supplementary Table S1.** Catalase activity and lipid peroxidation (TBARS) in unstressed and stressed yeast cells with 2 mM of H_2_O_2_, growing with and without 5 μM of melatonin (MEL). The strains used were: *S. cerevisiae* (BY4742, BY4741, Sigma 1278b, QA23, VIN 7, SC20, SB20 and Uvaferm HPS), *T. delbrueckii* (TdB and Tdp), *M. pulcherrima* (MpF and Mpp), *C. zemplinina* (Cz4 and Cz11) and *H. uvarum* (Hu4 and Hu35). Different letters in superscripts indicate significant differences between the conditions, p<0.05.

|  | **Catalase** (U catalase/ mg protein) | | | | **TBARS** (nmol MDA/ mg protein) | | | |
| --- | --- | --- | --- | --- | --- | --- | --- | --- |
|  | **Control** | **MEL** | **MEL H_2_O_2_** | **H_2_O_2_** | **Control** | **MEL** | **MEL H_2_O_2_** | **H_2_O_2_** |
| **BY4742** | 69.86 ± 4.71^a^ | 81.93 ± 4.18^b^ | 103.91 ± 2.18^c^ | 153.02 ± 1.25^d^ | 1.35 ± 0.01^a^ | 1.40 ± 0.01^b^ | 1.93 ± 0.12^c^ | 2.32 ± 0.07^d^ |
| **BY4741** | 80.67 ± 1.11^a^ | 95.42 ± 1.74^b^ | 110.97 ± 8.46^c^ | 159.28 ± 1.68^d^ | 1.15 ± 0.05^a^ | 1.25 ± 0.05^a^ | 1.98 ± 0.07^b^ | 2.23 ± 0.12^c^ |
| **Sigma 1278b** | 80.95 ± 1.79^a^ | 97.40 ± 2.71^b^ | 113.34 ± 8.93^c^ | 137.40 ± 6.59^d^ | 1.21 ± 0.05^a^ | 1.33 ± 0.08^a^ | 1.56 ± 0.07^b^ | 1.85 ± 0.09^c^ |
| **QA23** | 53.18 ± 1.01^a^ | 60.83 ± 0.86^b^ | 72.12 ± 5.87^c^ | 88.17 ± 5.38^d^ | 0.71 ± 0.07^a^ | 0.87 ± 0.11^a^ | 1.14 ± 0.11^b^ | 1.34 ± 0.04^c^ |
| **VIN 7** | 64.02 ± 5.03^a^ | 78.32 ± 6.67^b^ | 101.90 ± 6.37^c^ | 134.38 ± 8.37^d^ | 1.08 ± 0.08^a^ | 1.19 ± 0.02^b^ | 1.62 ± 0.10^c^ | 1.97 ± 0.06^d^ |
| **Uvaferm HPS** | 52.16 ± 1.43^a^ | 59.74 ± 0.75^b^ | 78.75 ± 8.00^c^ | 91.94 ± 2.06^d^ | 1.05 ± 0.08^a^ | 1.01 ± 0.06^a^ | 1.22 ± 0.05^b^ | 1.41 ± 0.11^c^ |
| **SC20** | 73.82 ± 4.93^a^ | 82.32 ± 2.60^b^ | 89.08 ± 6.32^b^ | 114.87 ± 8.90^c^ | 0.98 ± 0.07^a^ | 1.12 ± 0.06^a^ | 1.40 ± 0.05^b^ | 1.87 ± 0.15^c^ |
| **SB20** | 73.20 ± 4.22^a^ | 82.42 ± 2.55^b^ | 88.37 ± 1.80 ^c^ | 100.58 ± 4.60^d^ | 1.11 ± 0.04^a^ | 1.14 ± 0.07^a^ | 1.58 ± 0.10^b^ | 1.83 ± 0.12^c^ |
| **TdB** | 89.77 ± 6.54^a^ | 103.69 ± 1.56^b^ | 168.62 ± 4.17^c^ | 184.35 ± 3.43^d^ | 0.59 ± 0.05^a^ | 0.60 ± 0.06^a^ | 0.78 ± 0.07^b^ | 1.06 ± 0.10^c^ |
| **Tdp** | 96.30 ± 9.99^a^ | 115.18 ± 4.79^b^ | 140.94 ± 7.32^c^ | 171.22 ± 17.13^d^ | 0.56 ± 0.05^a^ | 0.64 ± 0.05^a^ | 0.78 ± 0.04^b^ | 1.09 ± 0.10^c^ |
| **MpF** | 92.56 ± 6.47^a^ | 99.48 ± 3.73^a^ | 143.26 ± 8.86^b^ | 175.67 ± 7.02^c^ | 0.47 ± 0.09^a^ | 0.48 ± 0.06^a^ | 0.67 ± 0.03^b^ | 0.77 ± 0.07^b^ |
| **Mpp** | 134.51 ± 10.91^a^ | 142.54 ± 6.34^a^ | 146.80 ± 7.13^a^ | 150.70 ± 1.93^a,b^ | 0.46 ± 0.10^a^ | 0.53 ± 0.08^a^ | 0.56 ± 0.12^a^ | 0.56 ± 0.08^a^ |
| **Cz4** | 90.33 ± 9.93^a^ | 91.78 ± 9.71^a^ | 82.46 ± 1.78^a^ | 94.64 ± 18.14^a^ | 0.42 ± 0.03^a^ | 0.47 ± 0.02^a^ | 0.58 ± 0.06^b^ | 0.62 ± 0.06^b^ |
| **Cz11** | 88.09 ± 7.00^a^ | 92.42 ± 4.99^a^ | 91.97 ± 2.96^a^ | 96.48 ± 1.16^a^ | 0.50 ± 0.04^a^ | 0.52 ± 0.08^a^ | 0.74 ± 0.13^b^ | 0.79 ± 0.06^b^ |
| **Hu4** | 88.80 ± 5.93^a^ | 112.16 ± 6.17^b^ | 143.85 ± 10.98^c^ | 171.55 ± 3.75^d^ | 0.91 ± 0.09^a^ | 0.94 ± 0.03^a^ | 1.22 ± 0.05^b^ | 1.42 ± 0.07^c^ |
| **Hu35** | 93.71 ± 1.28^a^ | 102.29 ± 2.88^b^ | 155.49 ± 5.32^c^ | 183.52 ± 10.27^d^ | 1.02 ± 0.06^a^ | 0.92 ± 0.09^a^ | 1.33 ± 0.07^b^ | 1.56 ± 0.05^c^ |
